# Correspondence

**Published:** 1910-10

**Authors:** D. B. Fields

**Affiliations:** San Francisco, Cal.


### Correspondence.

SAN FRANCISCO, CAL., September 8, 1910.

*Dr. F. E. Daniel, Editor of the Texas Medical Journal, Austin, Texas.*

DEAR SIR: I send you the following item of medical news regarding Dr. H. C. McClenahan, formerly of Texas, but now of San Francisco, Cal., which no doubt will be of interest to his many medical friends of your State. Dr. McClenahan, while in Texas, was assistant physician at the State Lunatic Asylum under Dr. B. M. Worsham for several years, resigning there to enter the army during the Spanish-American war, as assistant surgeon and captain of the First Texas Cavalry, U. S. V., under Col. L. R.

Hare. He removed to California in 1903, and was for six years assistant superintendent of the Gardner Sanitarium, at Belmont, California, an institution near San Francisco for nervous and mental diseases. Leaving there in August, 1909, and spending a year in Philadelphia and Europe for work in his special line, Dr. McClenahan returned in July and opened offices in San Francisco, and on August 15th was elected Assistant in Clinical Neurology, also Associate to the Chair of Legal Medicine and Psychiatry at the Cooper Medical College, which has lately been affiliated with the Leland Stanford, Jr., University, at its Medical Department. I am, Very turly yours,

D. B. FIELDS, M. D.

